# Sevoflurane causes greater QTc interval prolongation in chronically hyperglycemic patients than in normoglycemic patients

**DOI:** 10.1371/journal.pone.0188555

**Published:** 2017-12-01

**Authors:** Seishi Kimura, Shinichi Nakao, Atsuhiro Kitaura, Tatushige Iwamoto, Kei Houri, Mayuka Matsushima, Shinichi Hamasaki

**Affiliations:** Department of Anesthesiology, Kindai University Faculty of Medicine, OsakaSayama, Osaka, Japan; University of Bern, University Hospital Bern, SWITZERLAND

## Abstract

QTc interval prolongation is a serious diabetic complication and increases mortality rate. Hyperglycemia inhibits the rapid component of delayed rectifier potassium channel currents (Ikr) and prolongs the QTc interval on electrocardiograms. Sevoflurane also inhibits the Ikr and causes QTc interval prolongation. In fact, torsade de pointes occurred in a patient with poorly controlled diabetes mellitus during sevoflurane anesthesia. We enrolled 74 patients, including 37 normoglycemic patients (glycated hemoglobin [HbA1c]: <6.5%) (NG group) and 37 chronically hyperglycemic patients (HbA1c: ≥6.5%) (HG group). Anesthesia was induced with 2 mg/kg propofol and 0.3 μg/kg/min remifentanil, and maintained with 2% sevoflurane in 40% O_2_ and 0.2–0.3 μg/kg/min remifentanil. The QT interval and Tp-e interval (from the peak to the end of the T wave) were measured before and at 5, 10, 30, 60, 90, and 120 min after the administration of sevoflurane and adjusted for the patient’s heart rate (QTc and Tp-ec, respectively). P-values of <0.05 were considered statistically significant. The QTc and the Tp-ec intervals of the two groups did not differ significantly before the administration of sevoflurane. The QTc interval gradually increased with time in both groups and was significantly longer than the baseline value at 10 min after the administration of sevoflurane in both groups. The QTc interval of the HG group was significantly longer than that of the NG group at 90 min and 120 min after the administration of sevoflurane. The Tp-ec interval was not affected by sevoflurane in either group.We have demonstrated that sevoflurane significantly prolongs the QTc interval, and that the extent of the prolongation is significantly greater in chronically hyperglycemic patients than in normoglycemic patients. Although Tp-ec is not affected by sevoflurane, it should be noted that the simultaneous blockade of potassium channels would increase the risk of arrhythmias.

## Introduction

Several studies have detected relationships between QTc interval prolongation, diabetic complications, and an increased mortality rate in adults [1, 2]. Indeed, hyperglycemia inhibits the rapid component of delayed rectifier potassium channel currents (Ikr) and induces QTc interval prolongation on electrocardiograms (ECG) [3, 4]. On the other hand, volatile anesthetics, such as sevoflurane, also inhibit the Ikr [5, 6] and induce QTc interval prolongation [7, 8]. It should be noted that hyperglycemia potentiates the properties of Ikr-blocking drugs *in vitro* [3], so simultaneous blockade of potassium channels would increase the risk of arrhythmia. Actually, there was a report about a case in which marked QTc interval prolongation was followed by torsade de pointes (TdP) in a patient with poorly controlled diabetes who was placed under sevoflurane anesthesia [9]. However, few studies have investigated the effects of sevoflurane on the QTc interval in chronically hyperglycemic patients.

The purpose of this study was to investigate the effects of sevoflurane on the QTc interval and the Tp-e interval, which is a surrogate marker of the ventricular transmural dispersion of repolarization, in chronically hyperglycemic patients.

## Methods

The study was carried out after obtaining institutional approval from the Kindai University Faculty of Medicine Human Subjects Review Committee (No. 23–152) and written informed consent from all the patients.

**Fig 1:**
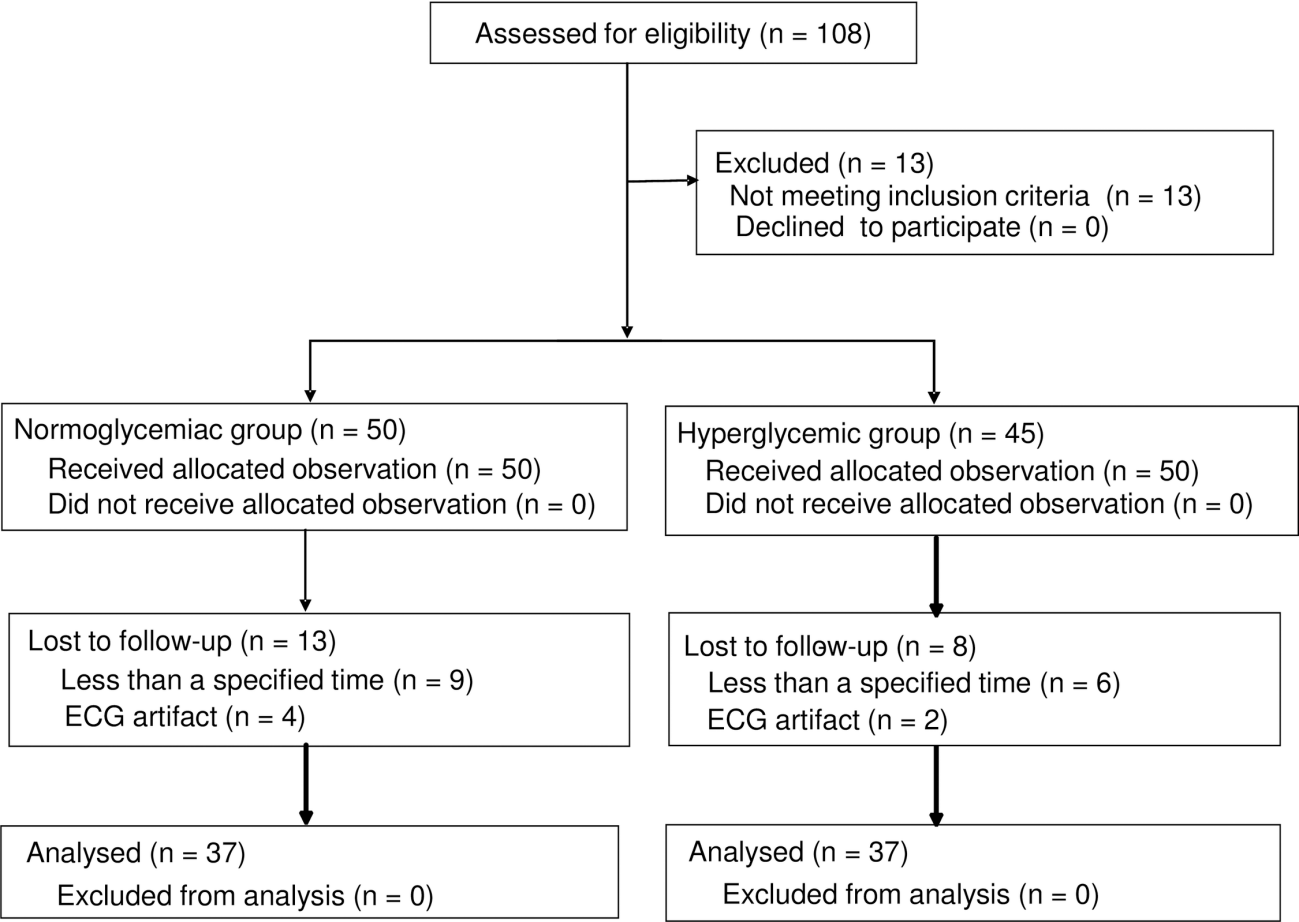
The structured patient flow chart for the experiment.

One hundred and eight patients aged 38–84 years old who underwent various kinds of elective surgeries, such as abdominal, otorhinolaryngological, gynecological, orthopedic, and urological surgeries, were recruited. The exclusion criteria included cardiac surgery; pregnant patients; and patients with severe ischemic heart disease, heart dysfunction, significant arrhythmias including atrial fibrillation and atrioventricular block, and/or a QTc interval of >480 ms, patients who had drugs known to inhibit Ikr and prolong the QTc interval, such as anti-arrhythmic drugs, psychotropic drugs, H2 blockers, and antibiotics (Fig 1).

Seventy-four patients were actually enrolled, and 37 patients whose serum hemoglobin A1c (HbA1c) concentrations (%), which represent long-term serum glucose levels, were ≥6.5% (HbA1c: ≥6.5%), were defined as the chronically hyperglycemic group (HG group), and 37 patients whose serum HbA1c concentrations were <6.5% (HbA1c <6.5%) were defined as the normoglycemic group (NG group). Their American Society of Anesthesiologists (ASA) physical statuses were I-III. In some patients, an epidural catheter was inserted, but it was not used until the measurements were completed.

In the operating room, at least standard monitors were installed, such as three-lead ECG (lead II), non-invasive blood pressure, the oxygen saturation of the peripheral artery (SpO_2_)_,_ end-tidal CO_2_ (EtCO_2_), and the bispectral index (BIS), and were recorded throughout the operation in all cases. General anesthesia was induced with 2 mg/kg propofol and 0.3 μg/kg/min remifentanil, and tracheal intubation was performed with the aid of 1 mg/kg rocuronium. Anesthesia was maintained with 2% sevoflurane in 40% O_2_ and 0.2–0.3 μg/kg/min remifentanil. When the patients’ blood pressure decreased (mean blood pressure <60 mmHg), ephedrine and/or phenylephrine were administered.

The items measured in this study included the RR interval, the QT interval (from the onset of the QRS complex to the end of the T wave), and the Tp-e interval (from the peak of the T wave to the end of the T wave) before the administration of sevoflurane (pre-anesthesia: T0), and at 5 (T1), 10 (T2), 30 (T3), 60 (T4), 90 (T5), and 120 (T6) min after the administration of sevoflurane. The end of T wave was defined as the intercept between the T wave and the isoelectric line with the tangent drawn through the maximum down slope of the T wave. Smaller U waves and those that are separate from the T wave were excluded. All measurements were conducted by two blinded observers. The QT interval was adjusted for the RR interval using Bazett’s formula (QTc = QT/[RR/1000]^1/2). Similarly, the Tp-e interval was also adjusted for the RR interval (Tp-ec = Tp-e/[RR/1000]^1/2).

The number of participants required for this study was determined using an f value of 0.25, as suggested by Cohen [10]; an α value of 0.05; and a power of 0.8. As a result, 74 patients were required for this study. Welch's t-test was used during comparisons of age, weight, or height, and the X^2^ test was used to assess the gender differences between the HG and the NG groups. Mann-Whitney U test was used for comparisons of HbA1c levels. The within-group changes in the QTc interval, the Tp-ec interval, heart rate (HR), and mean arterial pressure (MAP) with time were analyzed using one-way analysis of variance for repeated-measures followed by the Tukey post-hoc test. Comparisons of the QTc interval, the Tp-ec interval, HR, and MAP between the two groups were performed using Welch's t test. Pearson's correlation coefficient test and simple regression analysis were used to analyze the correlation between the QTc interval and the HbA1c level. Data are presented as mean ± SE or median (interquartile range) values, and P-values of <0.05 were considered to be statistically significant. The data were analyzed with GraphPad Prism 6.01 for Windows (GraphPad Software, Inc., La Jolla, CA).

## Results

Six patients who had been diagnosed with diabetes mellitus were included in the NG group because their HbA1c levels were <6.5%. No critical arrhythmia, such as TdP or ventricular fibrillation, occurred and no electrolyte abnormalities were observed during surgeries There were no significant differences in age, gender, weight, body mass index, or height between the HG and the NG groups, but both the HbA1c and blood glucose levels of the HG group were significantly higher than those of the NG group (Table 1).

**Table 1:** Baseline characteristics of the study population.

	Normooglycemic group	Hyperglycemic group	P value
	(NG group)	(HG group)	
	(n = 37)	(n = 37)	
Age (yr)	64.6 ±1.87	68.6 ±1.38	0.09
male	22	29	0.079
female	15	8	
Weight (kg)	57.6 ±1.83	61.9 ± 2.36	0.164
Height	160.9 ±1.67	163.4 ±1.55	0.279
Body mass index (kg/cm2)	22.1 ± 0.47	23.1 ±0.71	0.274
HbA1c(%)	5.9 (5.5–6.15)	7.4 (6.9–8.8)	<0.001*
Potassium (mEq/L)	4.29 ± 0.06	4.47 ± 0.07	0.05
Glucose (mg/dl)	112.1 ± 4.19	129.9 ± 4.79	0.007*
Pre-anesthesia QTc interval (ms)	402 ± 3.42	412 ± 4.54	0.07
Pre-anesthesia Tp-e interval(ms)	65.70 ± 2.10	72.1 ± 2.74	0.07
Type of surgeries	Otorhinolaryngological (15)	Otorhinolaryngological (8)	
	Urological (2)	Urological (6)	
	Gynecological (3)	Gynecological (1)	
	Orthopedic (2)	Orthopedic (1)	
	Abdominal surgery (15)	Abdominal (21)	
Drugs	Antihypertensive drug (8)	Antihypertensive drug (15)	
	Levothyroxine (3)	Levothyroxine (1)	
	Beta blocker (2)	Beta blocker (3)	
	Oral hypoglycemic drugs (2)	Oral hypoglycemic drugs (24)	
		Insulin (18)	

Data are presented as mean ± SE or median (Interquartile Range)

* P<0.05 vs NG group

**Fig 2:**
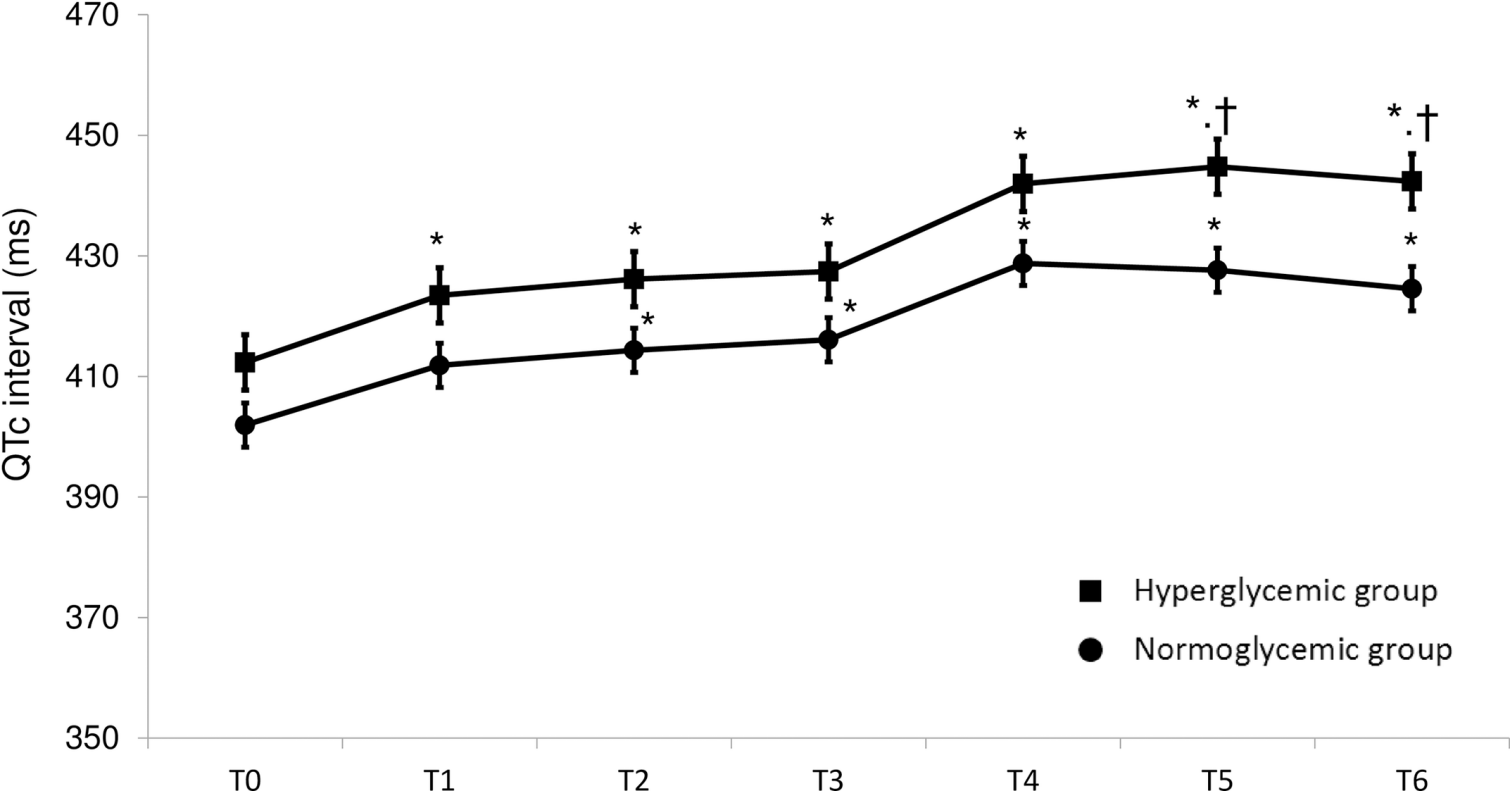
Mean QTc intervals (± SE; milliseconds) versus measurement time points before and after sevoflurane administration in the chronically hyperglycemic group (closed squares) and in the normoglycemic group (closed circles). The values recorded before the administration of sevoflurane (T0) and at 5 (T1), 10 (T2), 30 (T3), 60 (T4), 90 (T5), and 120 (T6) min after the administration of sevoflurane are shown. *P <0.05 compared with the baseline (pre) value; †P <0.05 compared with the normoglycemic group.

Before the administration of sevoflurane, there were no significant differences in the QTc or the Tp-ec interval between the HG and the NG groups. All of the QTc intervals recorded at ≥10 min after the administration of sevoflurane in the NG group were significantly longer than the baseline value (pre). In the HG group, all of the QTc intervals recorded at ≥5 min after the administration of sevoflurane were significantly longer than the baseline value. The QTc intervals recorded at 90 min (444.8 ± 5.76 vs. 427.6 ± 3.97 ms, P = 0.017) and 120 min (442.3 ± 6.05 vs. 424.5 ± 4.14 ms, P = 0.018) after the administration of sevoflurane in the HG group were significantly longer than those observed in the NG group (Fig 2).

**Fig 3:**
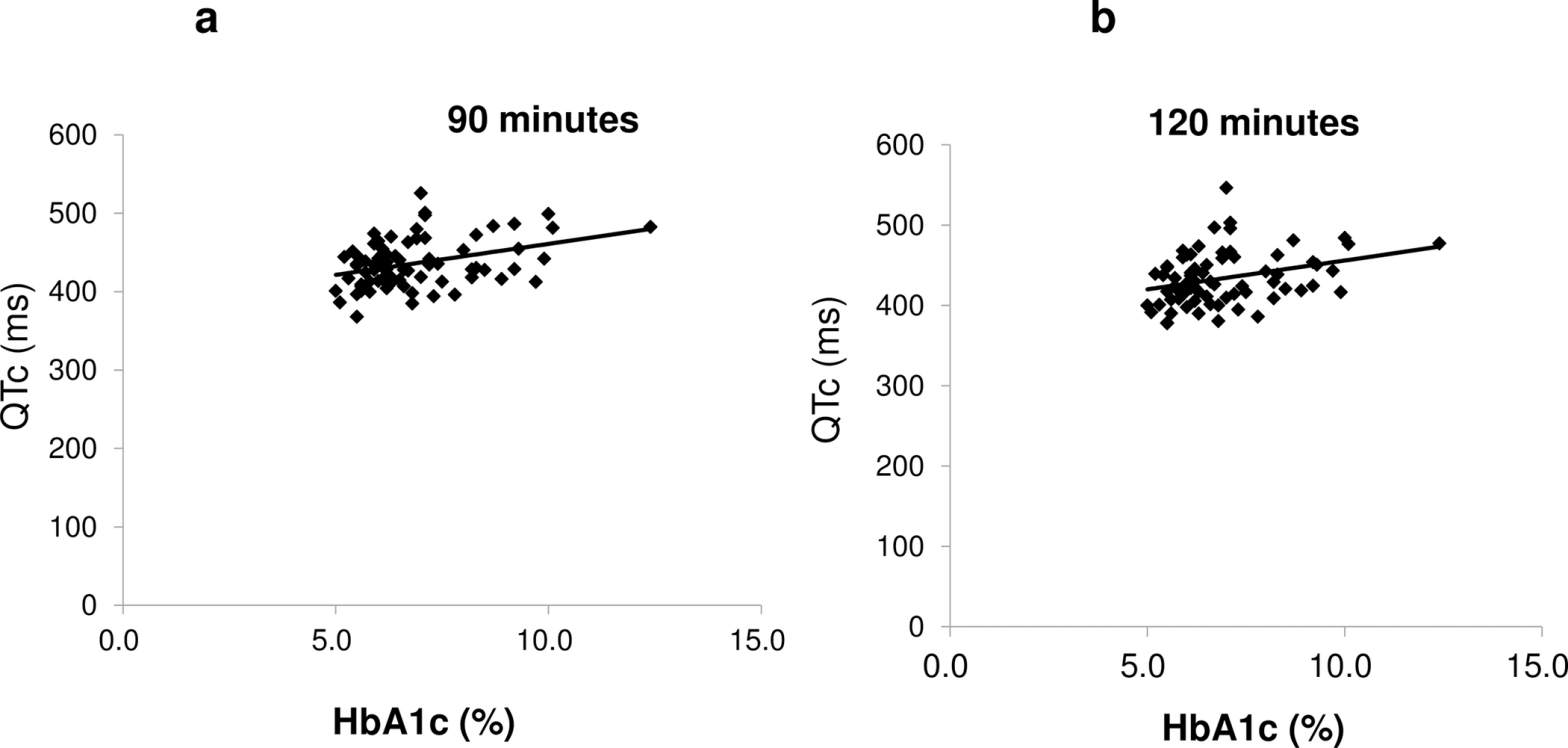
**3A and 3B. Correlation between the QTc intervals and HbA1c levels at 90 min (Fig 3A) or 120 min (Fig 3B) after sevoflurane administration.** 3a. The QTc intervals (Y-axis) recorded at 90 min after the administration of sevoflurane are plotted against HbA1c% values (X-axis). The formula for the regression line was Y = 7.910 X + 381.8 (solid line), and the coefficient ofdetermination (r2) was 0.1354 (P = 0.001). 3b. The QTc intervals (Y-axis) recorded at 120 min after the administration of sevoflurane are plotted against HbA1c% values (X-axis). The formula for the regression line was Y = 7.191X + 384.0 (solid line), and the coefficient of determination (r2) was 0.1013 (P = 0.006).

A linear regression analysis of the HbA1c level vs. the QTc interval showed good positive correlations at 90 min and 120 min after the administration of sevoflurane; the correlation coefficients at 90 and 120 min after the administration of sevoflurane were 0.3667 (P = 0.001) and 0.3183 (P = 0.006), respectively (Fig 3A and 3B).

**Fig 4:**
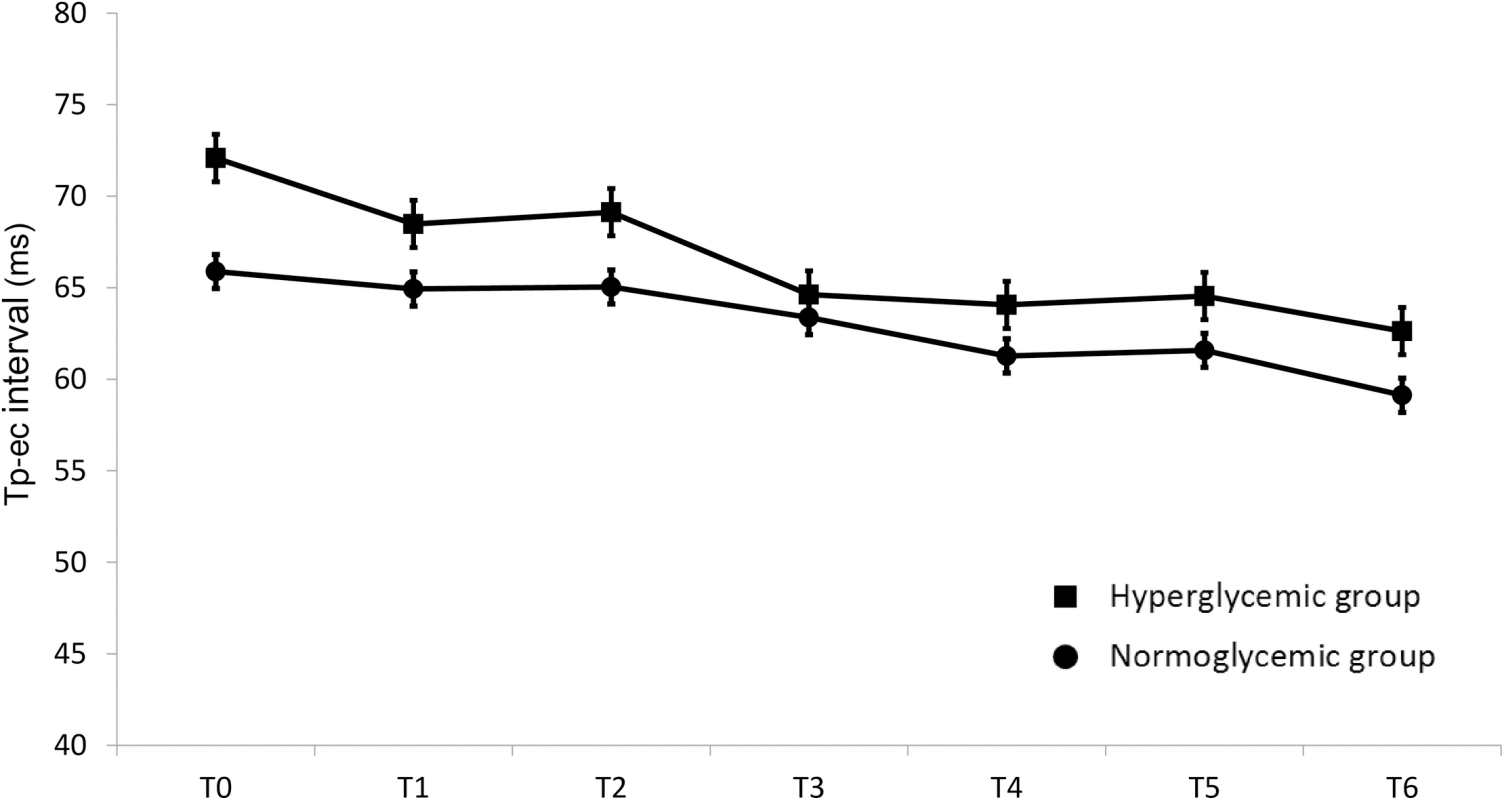
Mean Tp-ec intervals (± SE; milliseconds) versus measurement time points before and after sevoflurane administration in the chronically hyperglycemic group (closed squares) and in the normoglycemic group (closed circles). The values recorded before sevoflurane administration (T0) and at 5 (T1), 10 (T2), 30 (T3), 60 (T4), 90 (T5), and 120 (T6) min after the administration of sevoflurane are shown. *P<0.05 compared with the baseline (pre) value.

In contrast, the Tp-ec interval was not affected by sevoflurane in either group (Fig 4).

**Fig 5:**
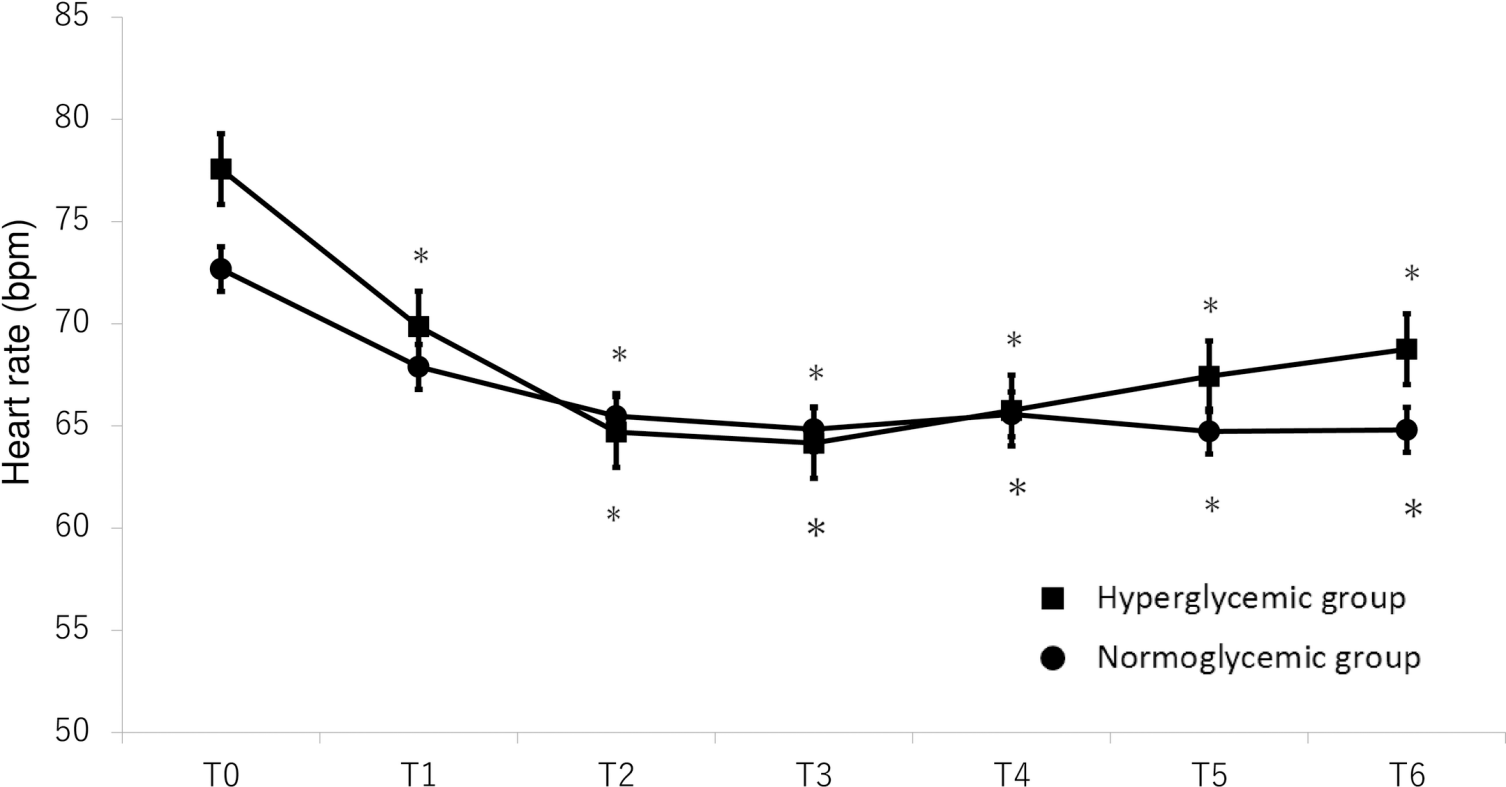
Mean heart rate (± SE; beats/min) versus measurement time points before and after sevoflurane administration in the chronically hyperglycemic group (closed squares) and in the normoglycemic group (closed circles). The values recorded before the administration of sevoflurane (T0) and at 5 (T1), 10 (T2), 30 (T3), 60 (T4), 90 (T5), and 120 (T6) min after the administration of sevoflurane are shown. *P<0.05 compared with the baseline (pre) value.

**Fig 6:**
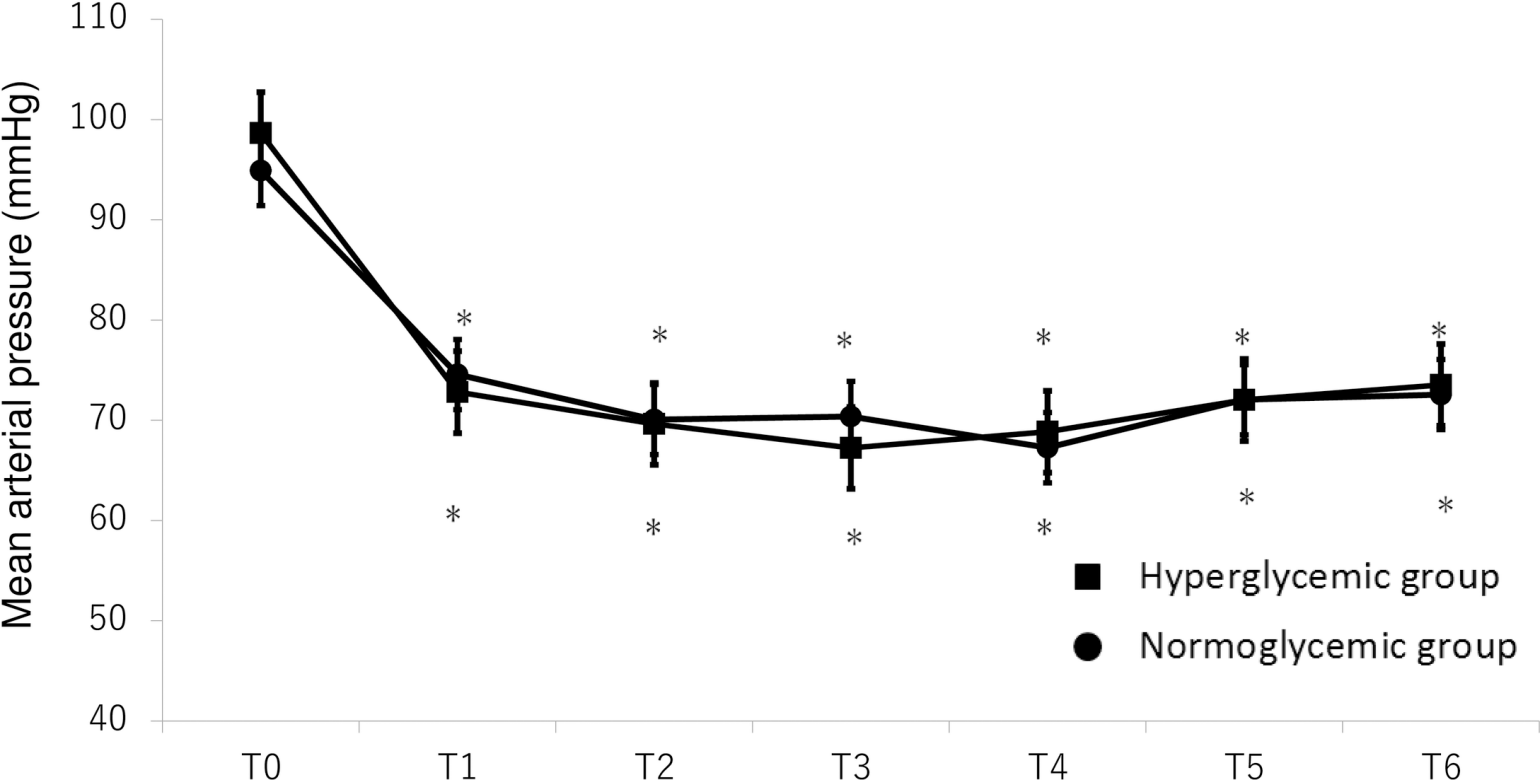
Mean arterial pressure (mean ± SE; mmHg) versus measurement time points before and after sevoflurane administration in the chronically hyperglycemic group (closed squares) and in the normoglycemic group (closed circles). The values recorded before the administration of sevoflurane (T0) and at 5 (T1), 10 (T2), 30 (T3), 60 (T4), 90 (T5), and 120 (T6) min after the administration of sevoflurane are shown. *P<0.05 compared with the baseline (pre) value.

Both heart rates and mean arterial blood pressure were significantly decreased after sevoflurane administration in either group but there were no significant differences of them between the HG group and the NG group (Figs 5 and 6).

## Discussion

We have confirmed that sevoflurane significantly prolongs the QTc interval [7, 8], and have demonstrated that sevoflurane induces significantly greater QTc interval prolongation in chronically hyperglycemic patients than in normoglycemic patients but does not affect the Tp-ec interval in either group.

Hyperglycemia inhibits Ikr and induces QTc interval prolongation [2–4]. Stern et al. [11] reported that the QTc interval was associated with the HbA1c concentration and autonomic function in diabetes patients. However, in the present study, there was no significant difference in the QTc interval between the HG and NG groups before the administration of sevoflurane, probably because the HG group included patients with slightly increased HbA1c levels. Actually, the mean QTc interval of the patients with HbA1c values of ≥8.0% was significantly longer than that of the NG group even before the administration of sevoflurane. Significant differences in the QTc intervals of the HG and NG groups and a significant positive correlation between the HbA1c level and the QTc interval were only observed at ≥90 min after the administration of sevoflurane, suggesting that it might take a certain amount of time for sevoflurane to induce marked QTc interval prolongation in patients with HG. Interestingly, Thiruvenkatarajan et al. [9] reported that TdP occurred 270 min after the administration of sevoflurane in a patient with poorly controlled diabetes mellitus (HbA1c: 9.2%), when both the QTc interval (from 450 ms before anesthesia to 690 ms) and the Tp-e interval (from 120 ms before anesthesia to 160 ms) were markedly prolonged.

Several studies have revealed that susceptibility to TdP arises from the induction of afterdepolarization and increased dispersion of ventricular repolarization, rather than QT interval prolongation *per se* [12]. The Tp-e interval is considered to be a surrogate marker of the ventricular transmural dispersion of repolarization, and Antzelevitch suggested that the Tp-e interval is a more reliable marker of TdP and/or lethal arrhythmias than the QTc interval [13]. Unlike the QT interval, there are still different opinions on whether the Tp-e interval is rate-dependent, and the corrected Tp-e (Tp-ec) interval is not as well-established as the QTc interval. However, we used the corrected Tp-e (Tp-ec) interval instead of the Tp-e interval because a recent study demonstrated that the Tp-e should also be corrected for heart rate [14].

It is generally recognized that sevoflurane is a safe anesthetic in terms of the risk of arrhythmia and that sevoflurane alone does not induce lethal arrhythmias, such as TdP, because it prolongs the QTc interval, but not the Tp-e interval, in patients without other risk factors for an increased QTc interval [7, 8]. However, it should be noted that the simultaneous blockade of potassium channels increases the risk of arrhythmia. Furthermore, as Tp-e interval prolongation only represents greater dispersion of transmural repolarization, which might increase the risk of transmural re-entry, it cannot be used to predict other risk factors for arrhythmia, such as afterdepolarization. In fact, TdP and/or lethal arrhythmias were induced under sevoflurane anesthesia in patients with long QT syndrome [15] or uncontrolled diabetes mellitus [9] or who were administered fluconazole [16], all of which suppress potassium channels and increase the QTc interval.

Several potential limitations of our study should be considered: First, we conducted the study in the clinical setting, and the anesthetic concentrations (e.g., 0.2–0.3 μg/kg/min remifentanil) were not strictly regulated, the sevoflurane concentration was not corrected on the MAC (minimum anesthetic concentration) basis for age, and the type of surgery was not restricted. In addition to sevoflurane, other anesthetics and anesthesia related drugs, such as propofol, remifentanil, rocuronium, ephedrine, and phenylephrine, were used. However, they were unlikely to affect the QTc interval and the Tp-ec interval directly, because they don’t seem to block Ikr, and furthermore, those drugs were used for all the patients and the concentrations of them were almost the same. Second, we used the Tp-ec interval instead of the Tp-e interval, which is not as well-established as the QTc interval.

## Conclusions

Sevoflurane prolonged the QTc interval to a greater extent in patients with chronic hyperglycemia than in normoglycemic patients. However, Tp-ec was not affected by sevoflurane in either group.

## Supporting information

S1 Dataset (XLSX)
